# What Factors Influence on Dermatology-Related Life Quality of Psoriasis Patients in South Korea?

**DOI:** 10.3390/ijerph18073624

**Published:** 2021-03-31

**Authors:** So Young Park, Kon Hee Kim

**Affiliations:** 1 Department of Nursing, Ewha University Medical Center, Seoul 07985, Korea; luttucedoll@gmail.com; 2 College of Nursing, Ewha Womans University, Seoul 03760, Korea

**Keywords:** depression, psoriasis, quality of life, stress, uncertainty

## Abstract

This descriptive study aimed to identify factors that can influence the quality of life of psoriasis patients. A total of 118 psoriasis outpatients completed a questionnaire consisting of the Dermatology Life Quality Index (DLQI), Psoriasis Life Stress Inventory (PLSI), Mishel Uncertainty in Illness Scale-Community form (MUIS-C), Center for Epidemiologic Studies-Depression scale (CES-D), and Self-Reported Severity Score (SRSS). The Psoriasis Area Severity Index (PASI) was calculated. The collected data were analyzed by descriptive statistics, *t*-test, one-way ANOVA, Scheffé test, Pearson’s correlation analysis, and stepwise multiple regression using SPSS/WIN 26.0. The average score of the DLQI was 14.19 ± 6.83 (range 0–30); the DLQI showed statistically significant differences according to age (F = 4.02, *p* = 0.021) and smoking type (F = 7.49, *p* = 0.001). The dermatology-related quality of life was significantly affected by psoriasis-related stress (β = 0.37, *p* < 0.001), depression (β = 0.35, *p* < 0.001), and subjective severity (β = 0.19, *p* = 0.005); these variables explained 60.7% of the variance in the dermatology-related quality of life (F = 61.34, *p* < 0.001). The results demonstrated that psoriasis-related stress, depression, and perceived severity of psoriasis should be considered when developing nursing interventions to improve patients’ quality of life.

## 1. Introduction

Psoriasis is a skin disease with >3% prevalence, with various environmental factors affecting its onset and course [1]. In Korea, >100,000 people have been diagnosed with psoriasis annually over the last decade, and the number of patients receiving this diagnosis has been steadily increasing [2]. Psoriasis is a chronic recurrent skin disease with an unknown etiology. Most patients with psoriasis need continuous treatment throughout their life, because the symptoms constantly fluctuate between improvement and exacerbation, depending on various factors, including immune disorders, infection, stress, drinking, and smoking [3,4]. In addition to the dermatological symptoms, psoriasis arthritis can develop in around 30% of psoriasis patients, increasing the vascular inflammation and cardiovascular disease incidences [5,6].

The main symptoms of psoriasis are scales and papules erythematosus, and even less-severe psoriasis can be harmful to patients, such that they experience severe stress due to low self-image and disturbed interpersonal relationships or socialization; this is particularly relevant in cases where lesions are located on exposed parts of the body [7]. Although scales and papules can appear across the entire body, they are frequently found on the elbows and knees; thus, from a cosmetic perspective, patients with psoriasis experience emotional stress due to exposed skin lesions. The characteristic symptoms of psoriasis can negatively influence the patients’ quality of life [8]. Furthermore, around 80% of psoriasis patients report stress as a factor that can worsen their symptoms; thus, emotional stress is a known causative and exacerbating factor [9].

Due to the characteristics of psoriasis, patients need to manage their symptoms continuously by controlling the exacerbating factors throughout their lives. The frequent relapses and unpredictable prognosis of the disease make prediction difficult and increase the uncertainty. Uncertainty in illness suggests an inability to determine the meaning of illness-related events and accurately anticipate or predict health outcomes [10]. The associated negative emotions can become an exacerbating factor, causing the periodic worsening of symptoms. A previous study reported that increased uncertainty could intensify depression [11]. In psoriasis, treatment and prognosis tend to be unpredictable and increase uncertainty in patients, which can result in negative emotions, consequently threatening their quality of life.

Quality of life is a complex and multidimensional concept and an index of subjective well-being in the mental, physical, and socioeconomic domains as perceived by individuals [12]. The quality of life associated with the health status and severity of the disease of patients is regarded as the health-related quality of life. This is an important indicator, as it influences the determination of the treatment goals, as well as the prognosis and disease management [13]. Interventions for improving the psychological health-related quality of life factors, such as depression and stress, and for improving the overall quality of life are crucial considering the high risks for depression, anxiety, and suicide in psoriasis. These risks are even higher in severe psoriasis [14]. Around 30% of dermatology outpatients have psychological disorders, and suicide attempts are twice as prevalent for patients who have severe psoriasis compared with those who do not [15,16].

Psoriasis patients’ mental health and quality of life can be negatively influenced by the sight of rapidly proliferating scales and skin lesions [16]. Additionally, they can have difficulties in controlling stress, drinking, smoking, and obesity [7]. Studies on nursing interventions for these problems are necessary; however, previous studies involving psoriasis patients are limited, because they mainly consist of case studies using various treatment methods [17,18] or survey reviews [19]. Although psoriasis treatment primarily involves long-term outpatient drug treatment, disease management at the patient-level is mainly based on information sharing among psoriasis-related interest groups.

Previous studies have reported that psoriasis can cause severe stress in patients. The higher the stress and uncertainty, the greater their depression and the worse their quality of life. The present study aimed to investigate the effect of psoriasis-related stress, uncertainty, depression, and psoriasis severity on patients’ quality of life and to provide data to support the potential nursing interventions for improving psoriasis patients’ quality of life.

## 2. Materials and Methods

### 2.1. Design

This study was a descriptive research to determine how the factors such as psoriasis-related stress, uncertainty, and depression influence the dermatology-related life quality.

### 2.2. Participants

The participants were patients >20 years old who were diagnosed with psoriasis and who received outpatient treatment at a university medical center in Seoul, Korea. Two-tailed test and multiple regression analysis were performed using G*Power program 3.1.9.2 with a medium effect size (f^2^ = 0.15), a power of 0.80, a significance level of 0.05, and a test variable of 10. The number of samples was 118. After the selection of 120 patients, a questionnaire was distributed. Two patients dropped out of the study, because they refused to participate; thus, the study was conducted with the remaining 118 participants. 

### 2.3. Measurements

#### 2.3.1. Psoriasis Severity

Psoriasis severity was measured using the Psoriasis Area Severity Index (PASI), an objective index, and the Self-reported Severity Score (SRSS), a subjective index. The PASI was calculated using Equation (1) and through the direct observation of psoriasis lesions. The score range was 0–72; a high score indicated a high level of symptom severity [17]. The score categories were mild (<5), moderate (5–15), and severe (≥15) [8]. The SRSS was measured on a linear scale from 0 (no symptoms) to 10 (most severe symptoms), which represents the patients’ perception of their severity; a higher score indicated more severe symptoms as perceived by the patients [20].

PASI = 0.1 × (E_head_ + I_head_ + S_head_) × A_head_ + 0.3 × (E_trunk_ + I_trunk_ + S_trunk_) × A_trunk_ + 0.2 × (E_arm_ + I_arm_ + S_arm_) × A_arm_ + 0.4 × (E_leg_ + I_leg_ + S_leg_) × A_leg_ (1)

E: Erythema, I: Induration, S: Scaling, and A: Area.

#### 2.3.2. Psoriasis-Related Stress

Psoriasis-related stress was measured using the Psoriasis Life Stress Inventory (PLSI), previously developed by Gupta and Gupta [21]. The PLSI is a four-point Likert scale with 15 items, each of which was assigned a value between 0 (strongly disagree) and 3 (strongly agree). The total scores ranged from 0 to 45; the higher the score, the more severe the stress. Scores < 10 indicated a low level of psoriasis-related stress, whereas scores > 10 indicated a high level of psoriasis-related stress. Cronbach’s α was 0.90 when the instrument was developed [21], and it was 0.91 in this study. 

#### 2.3.3. Uncertainty

To measure the level of uncertainty, a Korean translation by Oh [22] of the Mishel Uncertainty in Illness Scale-Community form (MUIS-C) [23] was used. The MUIS-C is a five-point Likert scale with 23 items; each item was assigned a value between 1 (strongly disagree) and 5 (strongly agree). The scores ranged from 23 to 115; the higher the score, the higher the level of uncertainty. Cronbach’s α was 0.74–0.92 [23] when developing the instrument, 0.75 when validated by Oh [22], and 0.78 in this study. 

#### 2.3.4. Depression

To measure depression, a Korean translation by Chon and Rhee [24] of the Center for Epidemiologic Studies–Depression Scale (CES-D) [25] was used. The CES-D is a four-point Likert scale with 20 items in the four subdomains: depressed feelings, positive feelings, interpersonal relationships, and physical degradation. Each item was assigned a value between 0 (strongly disagree) and 3 (strongly agree). The scores ranged from 0 to 60; scores > 16 indicated depression, and scores > 25 indicated major depression. Cronbach’s α was 0.85 [25] when developing the instrument, 0.89 when validated by Chon and Rhee [24], and 0.92 in this study. 

#### 2.3.5. Dermatology-Related Life Quality

The dermatology-related life quality was measured using the Korean version of the Dermatology Life Quality Index (DLQI) developed by Finlay and Khan after receiving permission from Cardiff University [26]. The DLQI is a four-point Likert scale with 10 items assigned a value between 0 (strongly disagree) and 3 (strongly agree). The minimum and maximum scores were 0 and 30, respectively; the higher the score, the lower the quality of life. The test–retest reliability coefficient was 0.99 when developing the instrument [26], and the Cronbach’s α was 0.91 in this study.

### 2.4. Data Collection

After the Institutional Review Board (IRB) approved this study, the data were collected from outpatients with psoriasis who visited the dermatology department at a university medical center between October and November 2018. We explained the study’s purpose and procedure to the patients when they visited the outpatient unit for treatment. Informed consent was obtained from those who agreed to participate in this study, and a structured questionnaire was distributed. The research staff read the survey aloud to those who could not fill out the form due to an impaired functional status. The PASI, an objective index of the severity level of psoriasis, was scored by the research staff when evaluating the patients’ lesions. A small token was offered to the participants for completing the survey. 

### 2.5. Data Analysis

Data analysis was performed using SPSS/WIN 26.0 (IBM, Armonk, NY, USA). The mean and standard deviation of the general characteristics, psoriasis-related stress, uncertainty, depression, and dermatology-related life quality of the participants were analyzed. The dermatology-related life quality was differentiated according to the participants’ general characteristics and analyzed using *t*-test and ANOVA; Scheffé test was used for the post-hoc analysis. Correlations between the psoriasis-related stress, uncertainty, depression, and dermatology-related life quality were determined by Pearson’s correlation analysis. The effects of psoriasis-related stress, uncertainty, and depression on the dermatology-related life quality were evaluated by multiple linear regression analysis.

## 3. Results

### 3.1. General Characteristics

The participants in this study were middle-aged and almost equally distributed by gender, and most of them were married and employed. Psoriasis onset was at 20–30 years old, and its duration was typically < five years. One-fifth of the participants spent 92–459 USD for psoriasis treatment over the last year. A quarter of the participants had a history of smoking, and most of them did not drink (Table 1).

### 3.2. Participants’ Psoriasis-Related Severity, Psoriasis Stress, Uncertainty, Depression, and Dermatology-Related Life Quality

Around one-third of the participants had moderate and severe psoriasis, according to the PASI. A subjective severity assessment indicated moderate psoriasis. Almost all of the participants had a high level of psoriasis-related stress, and most of them had mild-to-major depression. The uncertainty level was moderate to severe (Table 2).

### 3.3. Differences in Dermatology-Related Life Quality According to General Characteristics

The participants’ dermatology-related life qualities were significantly different for different ages (F = 4.02, *p* = 0.021), smoking types (F = 7.49, *p* = 0.001), PASI scores (F = 13.09, *p* < 0.001), PLSI scores (t = 6.17, *p* < 0.001), and CES-D scores (F = 32.00, *p* < 0.001) (Table 3).

### 3.4. Correlations among Psoriasis Stress, Uncertainty, Depression, and Dermatology Life Quality

The participants’ dermatology-related life qualities were negatively correlated with age (r = −0.311, *p* = 0.001). All scores, i.e., PASI (r = 0.32, *p* < 0.001), SRSS (r = 0.52, *p* < 0.001), PLSI (r = 0.74, *p* < 0.001), MUIS-C (r = 0.41, *p* < 0.001), and CES-D (r = 0.70, *p* < 0.001), were positively correlated with the DLQI (Table 4). 

### 3.5. Factors Influencing Dermatology-Related Life Quality

A multiple regression analysis was performed to identify the factors that could affect the participants’ dermatology-related life qualities, with significantly correlated variables as the predictors. We used objective severity, subjective severity, psoriasis-related stress, uncertainty, and depression as the predictive variables, as their significant correlations have been demonstrated. Of the general characteristics, age was chosen, as it showed a significant association with the dermatology-related life quality (Table 5). The detection of the multicollinearity and residual outliers was performed to test the independence of the predictive variables. The tolerance was 0.45–0.74, and the variance inflation factor (VIF) was 1.33–2.17, indicating that there was no multicollinearity issue. 

The factors influencing the participants’ dermatology-related life qualities were psoriasis-related stress (β = 0.37, *p* < 0.001), depression (β = 0.35, *p* < 0.001), and subjective severity (β = 0.19, *p* = 0.005). These variables could explain up to 60.7% of the variance in the dermatology-related life quality (F = 61.34, *p* < 0.001), and psoriasis-related stress and depression were the most significant factors based in the DLQI.

## 4. Discussion

In this study, we investigated the dermatology-related life qualities of psoriasis outpatients and identified the factors that can influence them. There were several relevant findings. First, there was a difference between the objective index and subjective index of the psoriasis severity. Second, the degree of psoriasis-related stress, uncertainty, and depression was medium or high among the psoriasis outpatients. Third, there was a significant difference in the dermatology-related quality of life according to the smoking type. Fourth, psoriasis-related stress, depression, and subjective severity were identified as significant influencing factors.

The psoriasis severity should be assessed objectively and subjectively, and it should be used as a clinical assessment tool to set treatment goals and select suitable treatment methods. Around 35% of the participants were assessed as having moderate and severe psoriasis based on the PASI, and the SRSS score was 5.08; a difference was observed between the objective index and subjective index of psoriasis severity. The results indicated that the patients’ subjective severity index could be severe regardless of the objective severity index. Therefore, the assessment and management of psoriasis, according to the subjective severity index, are crucial for improving psoriasis patients’ quality of life. A previous study reported the SRSS score as a significant factor that can influence the quality of life [27]. 

This study showed that the levels of psoriasis-related stress, uncertainty, and depression were medium or high for psoriasis patients. The psoriasis-related stress level was much higher than the level reported in previous studies [22,27,28]. These results indicated that restrictions on social activities due to a cycle of improving and worsening psoriasis can be perceived as stressful by the participants—87.3% of the participants had jobs that required social interactions. Therefore, nursing interventions should aim to understand and manage the psychosocial stress of psoriasis patients. The uncertainty level was also higher than that in previous studies [29,30,31], exceeding the uncertainty score of stomach cancer patients by >10 points. This suggests that psoriasis is a chronic and critical disease similar to cancer or cardiovascular disease, which develops on exposed areas such as the extremities and increases the uncertainty associated with the frequency of recurrence and unpredictable disease prognosis. Considering that uncertainty is a cognitive state attributed to a knowledge deficit [10], educational interventions are needed to improve patient understandings of psoriasis disease characteristics, treatment, and prognosis. In this study, around two-thirds of the participants belonged to the depression group (CES-D score > 16), exceeding the proportions in previous studies (around 50% of psoriasis patients with depression) [28,32]. This suggests that psoriasis patients should be monitored for depressive symptoms, because depression can develop due to the embarrassment of skin lesions; depressive symptoms may vary from mild symptoms such as a loss of motivation and low mood to suicide [33]. Taken together, nursing interventions are urgently needed for the assessment and mitigation of depression in psoriasis patients. 

The level of the participants’ dermatology-related life qualities was medium but still exceeded that in previous studies [28,34,35,36]. The higher the DLQI score, the lower the perceived quality of life [26]; thus, it is crucial to devise strategies to improve psoriasis patients’ quality of life. Our study showed that there was a significant difference in the dermatology-related life quality according to several general characteristics; particularly, there was a significant difference in the quality of life according to the smoking type. The DLQI was higher in the group of current smokers than in the group with no history of smoking. According to a previous study [37], smoking was more prevalent among psoriasis patients, and PASI scores were higher for smokers than for nonsmokers. Psoriasis patients’ quality of life should be improved by developing a health promotion program and using it to help patients cease smoking. These efforts are important, considering that psoriasis lesions and severity can worsen with an increase in daily smoking [37] and that depression can lead to smoking, which can exacerbate psoriasis and compromise the quality of life. 

This study identified psoriasis-related stress, depression, and the subjective index of psoriasis severity as significant influencing factors; these variables’ explanatory power was 60.7%. The participants’ dermatology-related life quality was high, with high levels of psoriasis-related stress, depression, and subjective severity. Previous studies have reported the age, fingernail lesion, health insurance, other accompanying diseases, and exercise habits as influencing factors; however, the explanatory power was less than 20% [34,38]. This study used psoriasis-related stress, depression, and subjective severity, which have not been studied previously as independent variables to confirm that psoriasis-related stress and depression could severely affect the dermatology-related life quality. The psoriasis-related stress level could explain the participants’ dermatology-related life qualities to the greatest degree, which is consistent with the previous findings [39] showing that psoriasis symptoms could cause deformities in the face, hair, and fingernails; psoriasis patients experience more difficulties in personal interactions due to esthetic impairment. These findings suggest that stress caused by psoriasis may be associated with a compromised psychosocial function in terms of interacting with others. Stress has been reported as a causative and exacerbating factor of psoriasis [40]. Based on previous studies [20,34,38] that reported the association of a low quality of life with a high level of stress, interventions such as stress management therapy should be developed to improve psoriasis patients’ quality of life. 

Depression was an influencing factor with the second-highest explanatory power. A systemic review of the literature on depression and suicide attempts found that psoriasis patients were diagnosed with depression three times the rate of healthy participants, and psoriasis was significantly correlated with depression even when other variables were controlled [33]. In the study by Ryu et al. [20], depression and psoriasis-related stress were identified as factors that could directly influence patients’ quality of life, which is consistent with our results. However, studies on psoriasis patients’ dermatology-related quality of life are limited. Nevertheless, considering that more than half of the study participants had a disease history of more than 10 years, previous studies on the quality of life of patients with chronic diseases were used for comparison. For patients with chronic liver disease, 65.7% of the total variance in the quality of life could be explained by depression [41]. For hemodialysis patients, depression was also identified as a key factor that could influence the quality of life, explaining 50.4% of the total variance [42]. The findings demonstrated that depression could affect the quality of life of patients with chronic diseases. Gupta et al. reported that the more severe the depression or subjective clinical symptoms, the higher the suicidal thoughts among psoriasis patients [43]. A high depression score is associated with severe pruritus. A previous study showed that reduced itching before and after treatment was associated with reduced depression scores [28]. Severe symptoms could inhibit emotional expression, intensify body dissatisfaction, worsen anxiety and depressive symptoms, and reduce the quality of life [44]. Therefore, psychological factors are important in the overall management of psoriasis patients. Additionally, an improved quality of life through depression management is expected to positively affect the psoriasis treatment process. This study demonstrated that subjective severity was also a significant factor that could influence the dermatology-related life qualities of psoriasis patients, similar to the findings of a previous study [20] showing that the subjective severity was significantly correlated with psoriasis patients’ quality of life, emotions, functions, symptoms, and social relationships. The level of subjective clinical symptoms evaluated by the patients themselves was noted to be an essential factor that could influence the quality of life. A study of patients with chronic autoimmune blister disease found that the dermatology-related quality of life was significantly lower with the higher disease severity, and it was high when the satisfaction with treatment was high, thus supporting our findings [18]. In addition to the objective index, our study also identified the subjective index of psoriasis severity as a factor that could influence the dermatology-related quality of life of psoriasis patients in the clinical assessment of psoriasis severity. Including the patients’ subjective assessment and medical staff’s objective assessment of clinical symptoms in treatment plans may improve patients’ quality of life by enhancing the treatment efficacy. Therefore, we propose an intervention study with a focus on differentiating physical problems from psychological problems according to psoriasis severity.

A number of studies have been conducted on psoriasis patients’ quality of life; however, those examining variables such as severity indices and personal health-related habits are limited. Therefore, future studies should investigate the overall status of the quality of life and influencing factors in addition to skin problems. Although we identified the factors that can affect psoriasis patients’ quality of life by evaluating the psychosocial variables, the assessment of the disease characteristics of psoriasis was limited in this study. In future studies, the disease characteristics (e.g., use of biological agents, location of lesions, and worsening period of symptoms) and lifestyle choices or habits (e.g., smoking and drinking) should be examined.

## 5. Conclusions

Based on our findings, psoriasis-related stress, uncertainty, and depression were significant factors that could influence psoriasis patients’ dermatology-related quality of life and worsen symptoms. Our study also demonstrated that self-reported severity was a significant factor that could influence psoriasis patients’ quality of life. Therefore, it is crucial to consider the patients’ perceptions about their disease when planning for nursing care. As indicated in previous studies, nursing interventions can effectively manage stress, depression, and uncertainty. Therefore, our findings would be useful for developing and implementing education programs for psoriasis patients. Additionally, the healthcare system should aim to manage patients’ psychosocial function by assessing the subjective and objective severity, psoriasis-related stress, and depression so that patients with psoriasis can lead healthy lives.

## Figures and Tables

**Table 1:** Participants’ general characteristics (*N* = 118).

Characteristics	Categories	N (%), Mean ± SD
Age		46.22 ± 13.37
	<29	19 (16.1)
	30–39	17 (14.4)
	≥40	82 (69.5)
Gender	Female	52 (44.1)
	Male	66 (55.9)
Marriage	Yes	85 (72.0)
	No	33 (28.0)
Occupation	Yes	86 (72.9)
	No	32 (27.1)
Education	≤High school	48 (40.7)
	≥College	70 (59.3)
Monthly income (U.S. dollar)	<1840	17 (14.4)
	1840–3680	43 (36.4)
	≥3680	34 (28.8)
	None	21 (20.3)
Drinking	Yes	43 (36.4)
	No	75 (63.6)
Smoking	Yes, currently smoking	34 (28.8)
	No, smoking in the past	31 (26.3)
	Never	53 (44.9)
Psoriasis onset age (year)		33.54 ± 14.18
	<20	17 (14.4)
	20–29	34 (28.8)
	30–39	31 (26.3)
	40–49	19 (16.1)
	≥50	17 (14.4)
Psoriasis duration (year)		13.68 ± 12.39
	<5.0	36 (30.5)
	5.0–9.9	17 (14.4)
	10.0–14.9	16 (13.6)
	15.0–19.9	17 (14.4)
	≥20	32 (27.1)
Psoriasis medical cost (U.S. dollar/year)	<92	18 (15.3)
	92–459	50 (42.4)
	460–919	33 (28.0)
	≥920	17 (14.4)
Body Mass Index (m/kg^2^)		23.57 ± 3.91
	<18.5	5 (4.2)
	18.5–22.9	55 (46.6)
	23.0–24.9	29 (24.6)
	≥25.0	29 (24.6)
Comorbidity	Yes	29 (24.6)
	No	89 (75.4)

**Table 2:** Psoriasis severity, psoriasis-related stress, uncertainty, depression, and dermatology-related life quality.

	Possible Range	Mean ± SD	Min	Max	*N* (%)
Psoriasis severity					
PASI ^1^		6.40 ± 6.28	0.20	44.20	
Mild					66 (55.9)
Moderate					37 (31.4)
Severe					15 (12.7)
SRSS ^2^	0–10	5.08 ± 1.92	1	10	
PLSI ^3^	0–45	18.83 ± 9.44	2	42	
Low					22 (18.6)
High					96 (81.4)
MUIS-C ^4^	23–115	69.04 ± 9.39	42	89	
CES-D ^5^	0–60	24.50 ± 11.53	0	48	
Normal					30 (25.4)
Mild depression					30 (25.4)
Major depression					58 (49.2)
DLQI ^6^	0–30	14.19 ± 6.83	0	29	

^1^ Psoriasis Area Severity Index. ^2^ Self-Reported Severity Score. ^3^ Psoriasis Life Stress Inventory. ^4^ Mishel Uncertainty in Illness Scale-Community form. ^5^ Center for Epidemiological Studies-Depression scale. ^6^ Dermatology Life Quality Index.

**Table 3:** Differences in the dermatology-related life qualities according to the general characteristics. (*N* = 118).

Characteristic		Dermatology Life Quality		
		Mean ± SD	*t*/F	*p* Scheffé
Age (year)	<29	16.26 ± 6.02	4.07	0.021
	30–39	17.35 ± 6.55		
	≥40	13.06 ± 6.81		
Gender	Female	13.77 ± 6.92	0.76	0.452
	Male	14.73 ± 6.73		
Marriage	Yes	13.68 ± 6.90	1.31	0.192
	No	15.52 ± 6.57		
Occupation	Yes	12.20 ± 8.38	1.21	0.227
	No	14.48 ± 6.57		
Education	≤High school	13.79 ± 6.76	0.06	0.814
	≥College	14.47 ± 6.91		
Monthly income (U.S. dollar)	<1840	12.52 ± 7.89	2.31	0.080
	1840–3680	16.32 ± 6.19		
	≥3680	13.23 ± 6.58		
	None	12.91 ± 6.92		
Drinking	Yes	15.25 ± 7.65	1.28	0.203
	No	13.58 ± 6.28		
Smoking	Yes, currently smoking	16.67 ± 6.50	7.49	0.001
	No, smoking in the past	10.58 ± 6.37		a > c
	Never	14.71 ± 6.52		
Psoriasis duration (year)	<5.0	14.08 ± 6.53	1.76	0.143
	5.0–9.9	15.82 ± 6.17		
	10.0–14.9	16.31 ± 6.29		
	15.0–19.9	15.29 ± 7.10		
	≥20	11.81 ± 7.27		
Psoriasis medical cost (U.S. dollar)	<92	11.94 ± 5.29	1.90	0.134
	92–459	13.44 ± 7.58		
	460–919	15.33 ± 5.91		
	≥920	16.58 ± 6.97		
Body Mass Index (m/kg^2^)	<23.0	14.86 ± 6.85	0.62	0.541
	23.0–24.9	13.72 ± 6.46		
	≥25.0	13.27 ± 7.20		
Comorbidity	Yes	14.24 ± 8.26	0.04	0.967
	No	14.18 ± 6.34		
PASI ^1^	Mild ^a^	12.18 ± 6.08	13.09	<0.001
	Moderate ^b^	14.97 ± 6.56		a,b < c
	Severe ^c^	21.13 ± 5.90		
PLSI ^2^	Low	7.14 ± 4.53	6.17	<0.001
	High	15.81 ± 6.22		
CES-D ^3^	Normal ^a^	8.93 ± 5.33	32.00	<0.001
	Mild depression ^b^	11.70 ± 4.98		a,b < c
	Major depression ^c^	18.21 ± 5.87		

^1^ Psoriasis Area Severity Index. ^2^ Psoriasis Life Stress Inventory. ^3^ Center for Epidemiological Studies-Depression scale.

**Table 4:** Correlations among the age, duration, psoriasis-related stress, uncertainty, depression, and dermatology-related life quality (*N S* = 118).

r(*p*)	Age	Psoriasis Duration	PASI ^1^	SRSS ^2^	PLSI ^3^	MUIS-C ^4^	CES-D ^5^	DLQI ^6^
Age	1	0.45 (<0.001)	−0.12 (0.202)	−0.23 (0.011)	−0.27 (0.003)	−0.27 (0.003)	−0.21 (0.022)	−0.31 (0.001)
Psoriasis duration		1	0.10 (0.913)	−0.01 (0.920)	−0.11 (0.252)	−0.34 (<0.001)	−0.12 (0.184)	−0.16 (0.084)
PASI ^1^			1	0.54 (<0.001)	0.35 (<0.001)	−0.07 (0.482)	0.28 (0.002)	0.32 (<0.001)
SRSS ^2^				1	0.52 (<0.001)	0.14 (0.145)	0.37 (<0.001)	0.52 (<0.001)
PLSI ^3^					1	0.39 (<0.001)	0.75 (<0.001)	0.74 (<0.001)
MUIS-C ^4^						1	0.45 (<0.001)	0.41 (<0.001)
CES-D ^5^							1	0.70 (<0.001)
DLQI ^6^								1

^1^ Psoriasis Area Severity Index. ^2^ Self-Reported Severity Score. ^3^ Psoriasis Life Stress Inventory. ^4^ Mishel Uncertainty in Illness Scale-Community form. ^5^ Center for Epidemiological Studies-Depression scale. ^6^ Dermatology Life Quality Index.

**Table 5:** Influencing factors on the dermatology-related life qualities among psoriasis outpatients (*N* = 118). VIF: variance inflation factor.

Variables	Dermatology Life Quality Index					
	B	β	*t*	*p*	Tolerance	VIF
(constant)	0.582		0.477	0.634		
PLSI ^1^	0.270	0.373	3.908	<0.001	0.368	2.719
CES-D ^2^	0.206	0.347	9.947	<0.001	0.433	2.309
SRSS ^3^	0.687	0.194	2.862	0.005	0.732	1.366
	R^2^ = 0.617, Adjusted R^2^ = 0.607, F = 61.337, and *p* < 0.001.					

^1^ Psoriasis Life Stress Inventory. ^2^ Center for Epidemiological Studies-Depression scale. ^3^ Self-Reported Severity Score.

## Data Availability

The data presented in this study are available on request from the corresponding author. The data are not publicly available due to participants’ privacy.
